# Ultrasound-Assisted Extraction of Bioactive Compounds from Strawberry Pomace: Optimization and Bioactivity Assessment

**DOI:** 10.3390/antiox15010050

**Published:** 2025-12-30

**Authors:** Milena Terzić, Biljana Lončar, Mirjana Petronijević, Sanja Panić, Aleksandra Cvetanović Kljakić, Jelena Arsenijević, Gokhan Zengin, Slavica Ražić

**Affiliations:** 1 Faculty of Technology Novi Sad, University of Novi Sad, Bulevar cara Lazara 1, 21000 Novi Sad, Serbia; milenavujanovic@uns.ac.rs (M.T.); mirjana.petronijevic@uns.ac.rs (M.P.); sanjar@tf.uns.ac.rs (S.P.); a.c.istrazivac@gmail.com (A.C.K.); 2 University of Belgrade–Faculty of Pharmacy, Vojvode Stepe 450, 11000 Belgrade, Serbia; jelena.arsenijevic@pharmacy.bg.ac.rs (J.A.); slavica.razic@pharmacy.bg.ac.rs (S.R.); 3 Science Faculty, Selcuk University, Campus Konya, Konya 42130, Turkey; biyologzengin@gmail.com

**Keywords:** strawberry pomace, greenness metric, artificial neural networks, phenolic compounds, by-product valorization

## Abstract

The growing production of strawberry-based foods generates large quantities of pomace, a phenolic-rich by-product with high valorization potential. This study aimed to optimize ultrasound-assisted extraction (UAE) of strawberry pomace and to evaluate the bioactivity of the resulting extracts. The greenness assessment using the AGREEprep metric yielded a score of 0.68/1, confirming the environmental friendliness of the process. Under the optimized UAE conditions (20 min, 50 °C, solid-to-liquid ratio 1:20 g/mL), the extract exhibited the highest total phenolic (16.49 mg GAE/g) and flavonoid contents (2.10 mg RE/g). The optimized extract showed strong antioxidant activity, with DPPH, ABTS, CUPRAC, and FRAP values of 32.70, 46.76, 57.29, and 38.90 mg TE/g, respectively. Enzyme inhibition was particularly pronounced against tyrosinase (55.45 mg KAE/g), while moderate inhibition of acetylcholinesterase and butyrylcholinesterase was also observed. Artificial neural network (ANN) modeling demonstrated excellent predictive performance (R^2^ > 0.99) and enabled effective optimization of extraction parameters. These findings confirm UAE as an efficient and sustainable approach for strawberry pomace valorization and highlight ANN-based optimization as a robust tool for developing multifunctional bioactive extracts for food, nutraceutical, and pharmaceutical applications.

## 1. Introduction

The global waste valorization market is rapidly expanding, driven by increasing sustainability demands, circular economy initiatives, and stricter environmental regulations. Valued at approximately $150 billion in 2025, the sector is expected to reach $250 billion by 2033 (CAGR 7%) [1]. Within this broader context, the fruit-processing industry produces substantial quantities of organic by-products. Nearly half of the 870 million tons of fruit produced worldwide in 2018 was processed into juices, generating waste streams that accounted for 20–80% of total fruit mass [2,3]. Although these by-products are rich in bioactive compounds, particularly phenolics, they remain largely underutilized.

Strawberry (*Fragaria × ananassa*) pomace, the major residue generated during juice and purée production, is especially noteworthy. It contains considerable levels of polyphenols, flavonoids, and other secondary metabolites associated with antioxidant, anti-inflammatory, and enzyme-inhibitory effects. The valorization of strawberry pomace aligns with circular bioeconomy principles, offering opportunities to reduce environmental impact while creating high-value functional ingredients [4,5].

In line with the increasing emphasis on waste valorization and sustainable resource use, strawberry by-products represent a promising and still underexploited raw material with significant potential for functional application [6].

Efficient recovery of these compounds requires advanced extraction technologies capable of maximizing yield while preserving bioactivity. Ultrasound-assisted extraction (UAE) has emerged as a green, rapid, and highly effective method, leveraging acoustic cavitation to enhance mass transfer, disrupt plant cell walls, and improve the penetration of extraction solvents. UAE allows reductions in processing time, energy consumption, and solvent usage, making it particularly suitable for heterogeneous plant matrices such as strawberry pomace. Optimal extraction requires precise control of parameters such as time, temperature, and sample-to-solvent ratio to ensure maximal recovery and retention of functional properties [7].

Despite the growing number of studies, challenges remain in standardizing extraction protocols, scaling up processes, and assessing economic feasibility for industrial applications. Artificial neural networks (ANNs) offer a powerful tool for modeling nonlinear interactions among extraction parameters, enabling accurate prediction and simultaneous optimization of multiple response variables, particularly when combined with multivariate analyses [8].

In this study, the valorization of strawberry pomace through UAE was systematically investigated, along with modeling and optimization using artificial neural networks (ANN). The extracts were comprehensively characterized for total phenolic and flavonoid content, antioxidant capacity, and inhibition of key enzymes, while multivariate analyses were employed to elucidate relationships among observed bioactivities. The primary objective was to identify UAE parameters that maximize the combined antioxidant and enzyme inhibitory potential of strawberry pomace extracts, providing a foundation for their application as high-value functional ingredients in food, nutraceutical, and pharmaceutical formulations. The distinct contribution of this study lies in an integrated approach, which combines ANN-based optimization with simultaneous assessment of multiple bioactivities and detailed phenolic profiling. In contrast to previous studies that address single bioactivities, provide limited characterization, or rely on conventional optimization methods, this work offers a broader evaluation of extract functionality and identifies UAE conditions that enhance bioactive potential, thereby facilitating the transition from laboratory-scale findings to prospective industrial application of strawberry pomace.

## 2. Materials and Methods

### 2.1. Chemicals and Reagents

All chemicals were purchased from Sigma-Aldrich (Darmstadt, Germany). The following reagents were used: 2,2′-azino-bis(3-ethylbenzothiazoline-6-sulphonic acid (ABTS), 1,1-diphenyl-2-picrylhydrazyl (DPPH), gallic acid, rutin, electric eel acetylcholinesterase (AChE) (type-VI-S, EC 3.1.1.7), horse serum butyrylcholinesterase (BChE) (EC 3.1.1.8), galantamine, acetylthiocholine iodide (ATChI), butyrylthiocholine chloride (BTChI) 5,5-dithio-bis(2-nitrobenzoic) acid (DTNB), tyrosinase (EC1.14.18.1, mushroom), glucosidase (EC. 3.2.1.20, from *Saccharomyces cerevisiae*), amylase (EC. 3.2.1.1, from porcine pancreas), sodium molybdate, sodium nitrate, sodium carbonate, Folin–Ciocalteu reagent, hydrochloric acid, Trolox, ethylenediaminetetraacetate (EDTA), neocuproine, cupric chloride, ammonium acetate, ferric chloride, 2,4,6-Tris(2-pyridyl)-s-triazine (TPTZ), ammonium molybdate, ferrozine, ferrous sulphate hexahydrate, kojic acid and acarbose. All chemicals were of analytical grade.

### 2.2. Material

The strawberry pomace used in this study was supplied by Nectar d.o.o. (Bačka Palanka, Serbia) as a by-product of their fruit processing facility. Afterward, it was transported to the laboratory and subjected to analysis. The initial moisture content of the strawberry pomace was 85% (on a wet basis). The obtained strawberry pomace samples were frozen and stored at −30 °C for 24 h, then subjected to lyophilization using the following device: Christ ALPHA 1–2 LD PLUS (Osterode am Harz, Germany). The lyophilization parameters were set as follows: pressure of 1.6 Pa, condenser temperature of −57 °C, shelf temperature at room temperature, and a process duration of 48 h. After lyophilization, the samples were finely ground into a powder of uniform particle size using a universal laboratory mill, type WZ-1 (Solem, ZBPP, Bydgoszcz, Poland).

### 2.3. Ultrasound-Assisted Extraction (UAE)

Strawberry pomace was extracted using an ultrasonic bath (Elma Schmidbauer GmbH, Konstanz, Germany) operating at a fixed frequency of 45 kHz. A measured mass of lyophilized strawberry pomace was transferred to a 150 mL Erlenmeyer flask, filled with a specified amount of water, and placed in the ultrasonic bath (Table 1). During the extraction, the flasks were fitted with ground-glass stoppers and subsequently reinforced with parafilm to prevent solvent evaporation. Various experimental parameters, such as extraction time, temperature, and sample to solvent ratio, were optimized. After extraction, the crude extract was filtered through 120 mm Whatman^®^ filter paper (Hangzhou, China) and collected in glass vials. Each experiment was performed in triplicate, and the extracts were stored at 4 °C until analysis.

### 2.4. Greenness Metric for Sample Preparation

**Figure 1:**
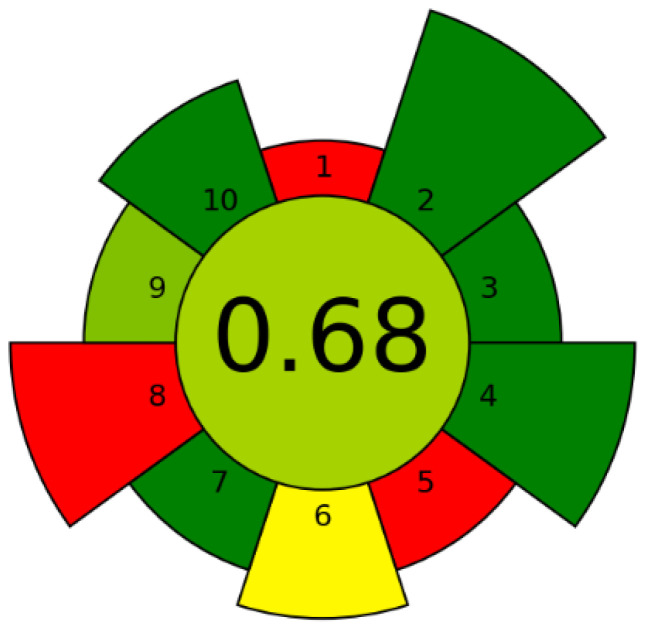
(Table S1, Supplementary Material). Greenness assessment of the sample preparation—AGREEprep pictogram.

To evaluate the environmental impact of the sample preparation method (lyophilization and extraction), AGREEprep (available at https://doi.org/10.1016/j.trac.2022.116553) was used as a greenness metric tool [9,10] before bioactivity testing. The main parameters considered were sample preparation location, use of hazardous materials, sustainability, mass of generated waste, sample size efficiency, sample throughput, energy consumption, and operational safety. The total AGREEprep score in this study was 0.68/1 (Figure 1), indicating that the method is environmentally friendly and does not negatively impact the environment. The red fields in the pictogram are due to the sample preparation being ex situ and the large amount of crude strawberry pomace subjected to lyophilization, which is a more energy-consuming step.

### 2.5. Determination of Total Phenolic (TPC) and Flavonoid Content (TFC)

The TPC of strawberry pomace extracts was determined using the Folin–Ciocalteu method (F9252, Merck, Darmstadt, Germany), while the TFC was measured using the aluminum chloride colorimetric method (AlCl_3_; 11019, Merck, Darmstadt, Germany) according to the procedures described elsewhere [11]. A detailed description of these analytical procedures is provided in the Supplementary Materials. These analyses were conducted in triplicate and presented as mean values.

### 2.6. Antioxidant and Enzyme Inhibitor Activity

The antioxidant capacity of strawberry pomace extracts was evaluated using in vitro assays, including DPPH, ABTS, CUPRAC, FRAP, metal chelating (MC), and total antioxidant activity (PM) [11,12]. Results were reported as mg Trolox equivalents (TE)/g extract for DPPH, ABTS, CUPRAC, and FRAP; mg EDTA equivalents (EDTAE)/g extract for MC; and mmol TE/g extract for PM. Enzyme inhibitory activities were measured against acetylcholinesterase (AChE), butyrylcholinesterase (BChE), tyrosinase, α-amylase, and α-glucosidase, using galanthamine, kojic acid, and acarbose as reference standards, with activities expressed as GALAE, KAE, and ACAE equivalents per g of extract, respectively [11,12]. A detailed description of these analytical procedures is provided in the Supplementary Materials. All analyses were conducted in triplicate.

### 2.7. Statistical Analysis

The statistical evaluation of the experimental results was carried out using StatSoft Statistica 10.0^®^ software [13]. For visualization purposes, a color plot diagram was created with R software v.4.0.3 (64-bit) [14].

### 2.8. ANN Modelling

**Figure 2:**
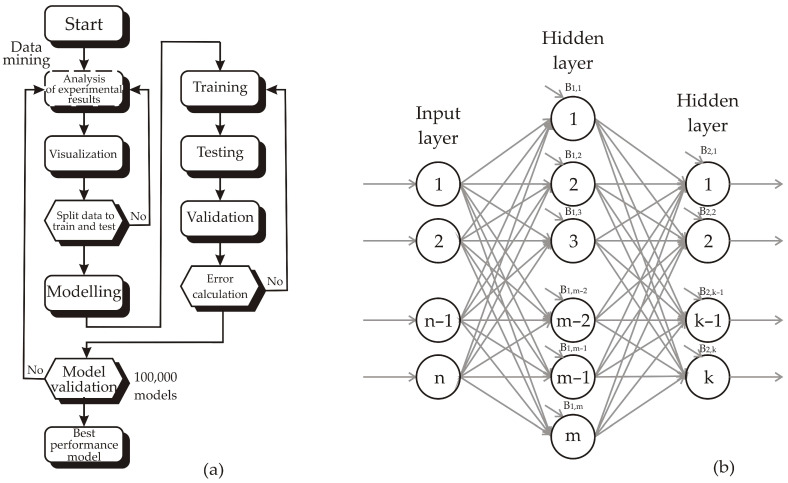
ANN structure: (**a**) Flowchart of the conducted research and (**b**) three layer structure.

Artificial neural network (ANN) models with strong capability for nonlinear function approximation were developed using a multi-layer perceptron (MLP) architecture, consisting of input, hidden, and output layers [15]. Before constructing the ANN models, normalization of both input and output datasets was performed to improve predictive efficiency [16]. The training procedure of the networks followed the methodology reported in the study [17]. Figure 2 illustrates the flowchart and the three-layer architecture (input, hidden, and output layers) of the study, designed to identify the most suitable ANN model based on both predictive performance and model error rates. To ensure robust validation despite the small dataset (N = 15), the observations were divided into training (60%), testing (20%), and validation (20%) sets, 100,000 models were calculated in order to find the optimal model constructions.

The weight parameters and biases for hidden and output layers are expressed as matrices and vectors W_1_ and B_1_, and W_2_ and B_2_, respectively. The ANN model can be mathematically described as:

(1) $$Y=f_{1}\left(W_{2}\cdot f_{2}\left(W_{1}\cdot X+B_{1}\right)+B_{2}\right)$$

where *Y* denotes the output matrix, *f*_1_ and *f*_2_ represent the transfer functions of the hidden and output layers, and *X* is the matrix of inputs [18].

The weight coefficients *W*_1_ and *W*_2_ were iteratively optimized during the learning cycle to minimize the deviation between predicted and experimental values, ensuring improved model accuracy [19]. Three ANN models were developed to foresee and optimize the parameters: ANN_1_ for TPC, TFC, ANN_2_ for DPPH, ABTS, CUPRAC, FRAP, MC, PM, and ANN_3_ for AChE, BChE, Tyrosinase, α-amylase, α-glucosidase, according to: time, temperature and plant to solvent ratio.

### 2.9. Model Validation

The performance of the ANN models were validated through several statistical indices, namely the coefficient of determination (r^2^), reduced chi-square (χ^2^), mean bias error (MBE), root mean square error (RMSE), and mean percentage error (MPE), sum of square error (SSE) and absolute average relative deviation (AARD), through the application of the following equations. These parameters were calculated using the following equations [20].

(2) $$\chi^{2}=\frac{\sum_{i=1}^{N}{\left(x_{\exp,i}-x_{pre,i}\right)}^{2}}{N-n}$$

(3) $$RMSE={\left[\frac{1}{N}\cdot \sum_{i=1}^{N}{\left(x_{pre,i}-x_{\exp,i}\right)}^{2}\right]}^{1/2}$$

(4) $$MBE=\frac{1}{N}\cdot \sum_{i=1}^{N}\left(x_{pre,i}-x_{\exp,i}\right)$$

(5) $$MPE=\frac{100}{N}\cdot \sum_{i=1}^{N}\left(\frac{\left|x_{pre,i}-x_{\exp,i}\right|}{x_{\exp,i}}\right)$$

(6) $$SSE=\sum_{i=1}^{N}{\left(x_{pre,i}-x_{\exp,i}\right)}^{2}$$

(7) $$AARD=\frac{1}{N}\cdot \sum_{i=1}^{N}\left|\frac{x_{\exp,i}-x_{pre,i}}{x_{\exp,i}}\right|$$

where x_exp,i_ marks the experimental values and x_pre,i_ present value obtained by the model, N and n are the number of observations and constants, respectively.

## 3. Results

### 3.1. Total Phenolic and Flavonoid Content

The total phenolic content (TPC) and total flavonoid content (TFC) of strawberry pomace extracts under different ultrasound-assisted extraction (UAE) conditions are presented in Table 2.

The TPC of the strawberry pomace extracts in this study ranged from 8.69 to 16.49 mg GAE/g, while TFC varied between 0.80 and 2.10 mg RE/g. Sample 6 (20 min extraction at 50 °C, sample-to-solvent ratio 1:20 g/mL) showed the highest TPC and TFC values, indicating that intermediate extraction time, moderate temperature, and higher solvent volume together ensure optimal recovery of phenolic and flavonoid compounds. In contrast, Samples 7 and 14, extracted under shorter times and/or lower solvent volumes, exhibited the lowest TPC and TFC, suggesting that these conditions limited the extraction efficiency of bioactive compounds. Lower TPC values observed in extracts obtained at higher temperatures (75 °C) may indicate temperature-related effects on the stability and recovery of phenolic compounds. Since specific degradation products were not the primary focus of this study, the observed trends should be interpreted as indicative of temperature-related effects on phenolic recovery rather than as direct evidence of phenolic degradation.

The TPC values measured in this study are in agreement with previously published reports [21,22,23]. Villamil-Galindo et al. [21] reported that TPC in strawberry by-products treated with UVA and extracted via UAE ranged from 7.1 to 13.5 g GAE/kg (equivalent to 7.1–13.5 mg GAE/g) [21]. Under their optimal conditions (1:30 ratio, 46% ethanol, 100% ultrasound power), the authors reported a TPC of 13 mg GAE/g, whereas the maximum TPC achieved in the present study was 16.49 mg GAE/g using water as the extraction solvent. It should be noted that direct quantitative comparison between studies is limited due to differences in extraction systems and operational parameters, including solvent composition, ultrasound mode (bath vs. probe), ultrasound frequency (45 kHz in the present study), power settings, and sample pretreatment. In another study, UAE extracts from mixed industrial fruit wastes exhibited substantially higher TPC values (86–133 mg GAE/g), which can be attributed not only to the heterogeneous nature of the raw material but also to differences in ultrasound equipment and process parameters. Nevertheless, the results obtained in this study remain highly relevant for the valorization of strawberry pomace under mild, water-based extraction conditions, while the reported TFC values (0.80–2.10 mg RE/g) provide complementary information not addressed in the cited studies. Moreover, the results of our study are in accordance with those obtained by UAE for fresh strawberry fruits using response surface methodology (yielding up to 18.78 mg GAE/g TPC and 10.52 mg CE/g TFC) [23], despite using industrial by-products rather than fresh fruits. In the present study, TFC values are expressed in rutin equivalents (RE), whereas in the cited study, they are expressed in catechin equivalents (CE). Considering the differences in molar mass, direct numerical comparisons between studies were indicative only. The lower TFC values observed in our study can therefore be attributed to the matrix difference, extraction solvent/system, and potentially reduced extractability of flavonoids from pomace.

Furthermore, the strong correlation observed between TPC and TFC across all samples aligns with previous findings that flavonoids constitute a substantial fraction of total phenolics in berry by-products [24,25,26]. Variations in extraction profiles can be attributed to differences in plant material, solvent composition, ultrasound intensity, solid-to-solvent ratio, and other process parameters. In any case, these observations confirm that the UAE conditions optimized in this study provide an effective balance between enhanced mass transfer and minimal thermal degradation, enabling maximal recovery of phenolic compounds and flavonoids from strawberry pomace.

### 3.2. Antioxidant Activity of Strawberry Pomace Extracts

The antioxidant capacity of strawberry pomace extracts under different UAE conditions was assessed using six complementary assays (DPPH, ABTS, CUPRAC, FRAP, metal chelation, and phosphomolybdenum), providing a comprehensive evaluation of their radical scavenging and reducing potential (Table 3).

The antioxidant activity of the strawberry pomace extracts exhibited substantial variability depending on the extraction parameters. Sample 6 (20 min, 50 °C, sample-to-solvent ratio 1:20 g/mL) showed the highest activity across all assays: DPPH 32.70 mg TE/g, ABTS 46.76 mg TE/g, CUPRAC 57.29 mg TE/g, FRAP 38.90 mg TE/g, MC 19.14 mg EDTAE/g, and PM 0.54 mmol TE/g. These trends were closely aligned with the observed total phenolic (TPC) and total flavonoid (TFC) contents, confirming that phenolics and flavonoids constitute major contributors to the antioxidant capacity of strawberry pomace extracts. Previous studies investigating the composition of strawberry pomace have reported that, in addition to phenolic compounds and flavonoids, the strawberry pomace contains other bioactive constituents, including organic acids (ascorbic, malic, citric, succinic, and quinic acids), anthocyanins (pelargonidin derivatives), ellagitannins, and fiber-associated polysaccharides [27,28,29]. These compounds may further contribute to the observed antioxidant activity, potentially through additive or synergistic effects, emphasizing the complex and multifactorial nature of the pomace’s antioxidant potential.

On the other hand, extraction under moderate conditions, intermediate temperature, extended contact time, and higher solvent volume facilitated efficient solubilization and diffusion of bioactive compounds from the pomace matrix. Conversely, harsher conditions, such as elevated temperatures (75 °C), may have compromised the stability of thermolabile antioxidants, particularly certain phenolic compounds, contributing to reduced recovery as reflected in lower TPC and TFC values. These observations underscore the importance of carefully balancing extraction parameters to maximize the yield of functionally active constituents [30].

While total phenolic (TPC) and flavonoid (TFC) contents are generally reliable indicators of reducing capacity, as measured by DPPH, ABTS, FRAP, CUPRAC, and PM assays, metal chelation (MC) activity reflects a mechanistically distinct antioxidant pathway, specifically the sequestration of transition metal ions such as Fe^2+^ and Cu^2+^. The relatively weaker association between MC activity and total phenolic content likely arises not only from the structural specificity of individual phenolic molecules but also from contributions of other bioactive constituents in the pomace, including organic acids, anthocyanins, ellagitannins, and fiber-associated polysaccharides [27,28,29]. Such interactions may produce additive or synergistic effects, underscoring the multifactorial nature of antioxidant activity in complex matrices. These observations highlight the importance of applying a complementary battery of assays to comprehensively assess the diverse mechanisms by which strawberry pomace extracts exert antioxidant effects.

Comparative studies on berry by-products corroborate these observations. UAE of strawberry and blueberry pomace has been demonstrated to markedly enhance radical scavenging and reducing activities relative to conventional extraction methods, highlighting the importance of optimized extraction parameters for effective antioxidant recovery [25,30,31].

The high content of phenolic molecules, in combination with the results of multiple complementary antioxidant assays, supports the notion that flavonoids comprise a substantial fraction of the phenolic pool in strawberry pomace. It should be acknowledged, however, that the phenolic profile provides information on antioxidant potential under in vitro conditions and does not necessarily predict in vivo efficacy, where factors such as bioavailability, metabolism, and cellular interactions play a decisive role. While these findings cannot yet be extrapolated directly to industrial applications, they provide a robust foundation for further investigations aimed at valorizing strawberry pomace as a potential source of natural antioxidants for food, nutraceutical, and pharmaceutical formulations.

### 3.3. Enzyme Inhibitor Activity of Strawberry Pomace Extracts

The enzyme inhibitory activities of strawberry pomace extracts obtained under different UAE conditions are summarized in Table 4.

The extracts were evaluated against key enzymes implicated in neurodegenerative disorders (acetylcholinesterase [AChE], butyrylcholinesterase [BChE]), hyperpigmentation (tyrosinase), and carbohydrate metabolism (α-amylase, α-glucosidase). Significant variability was observed across samples, indicating a strong influence of extraction parameters on the recovery of bioactive compounds responsible for enzyme inhibition.

Notably, the highest AChE inhibitory activity was observed in sample 3 (2.54 mg GALAE/g), while the highest BChE inhibition was detected in sample 7 (2.36 mg GALAE/g). Tyrosinase inhibition peaked in sample 6 (55.45 mg KAE/g). In contrast, α-amylase and α-glucosidase inhibitory activities were generally moderate, with the maximum α-amylase inhibition in sample 11 (0.902 mmol ACAE/g) and α-glucosidase inhibition again highest in sample 3 (1.114 mmol ACAE/g). These results indicate that the UAE conditions optimized for TPC, TFC, and antioxidant activity (Sample 6) do not coincide with the optimal conditions for all enzyme inhibitory activities, emphasizing that each enzymatic response is influenced by specific phytochemical constituents.

When comparing with the literature, it is evident that there are relatively few studies dealing with the enzyme-inhibitory activity of fruit-pomace extracts obtained by UAE. For example, a recent investigation on grape pomace reported AChE-inhibitory compounds such as kaempferol-3-O-glucoside using a green extraction technique [32]. Similarly, a review of food-derived AChE inhibitors highlighted that polyphenolic-rich extracts can act as cholinesterase inhibitors, but these studies seldom address multiple enzyme systems simultaneously or optimized extraction parameters [33,34,35,36]. Recent reports on the enzyme-inhibitory activity of fruit pomace extracts obtained via UAE remain limited, but the existing literature demonstrates the substantial bioactive potential of such residues. Laaraj et al. reported that Soxhlet extracts of Punica granatum peel inhibit α-glucosidase and α-amylase, highlighting the enzyme-inhibitory capacity of phenolics such as ellagic acid and catechin, even without UAE [37]. Fermentation and enzymatic treatment of fruit residues have similarly been shown to enhance the release of bioactive compounds, thereby affecting enzyme inhibition [38]. Raspberry pomace incorporated into apple-based products exhibited notable multi-enzyme inhibitory potential, underscoring the functional relevance of polyphenols in these matrices [39]. Lingonberry and grape pomace extracts further demonstrated pronounced inhibitory effects against multiple enzymes, confirming the enzymatic bioactivity of fruit by-products [40]. These studies, although not directly utilizing UAE, provide a critical context for evaluating enzyme-inhibitory activity and underscore the importance of applying optimized UAE to fully exploit the bioactive potential of fruit pomace. The scarcity of studies that optimize UAE while simultaneously evaluating multiple enzyme inhibitors in pomace matrices underscores the novelty and contribution of the present work.

Generally, our findings demonstrate that the optimized UAE parameters (moderate temperature, sufficient extraction time and elevated solvent volume) not only enhanced phenolic and flavonoid recovery but also resulted in inhibitory activities across several enzyme classes. This dual focus on extraction optimization and multi-enzyme inhibitory assessment provides a valuable contribution to the valorization of strawberry pomace as a multifunctional ingredient for neuroprotective, anti-hyperpigmentation, and antidiabetic applications.

**Figure 3:**
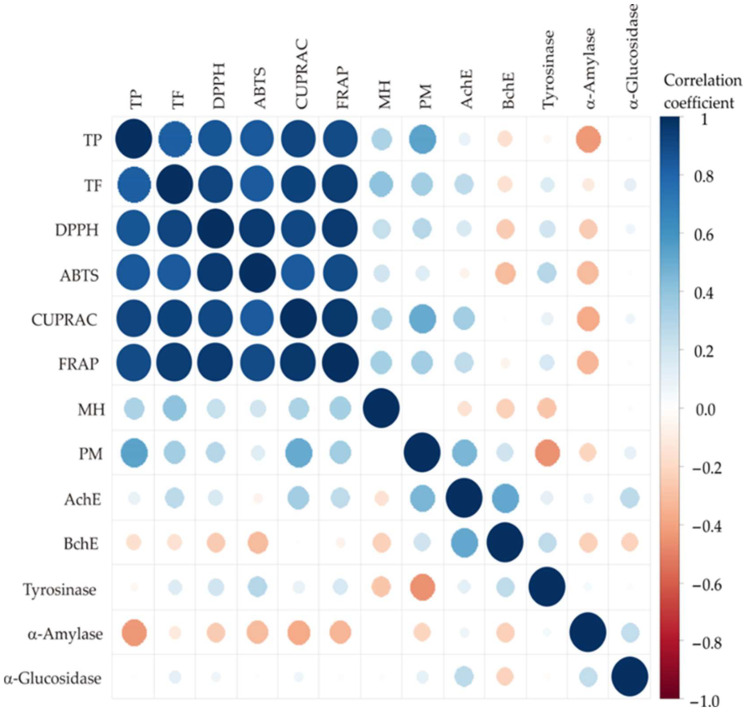
The correlation analysis between observed responses.

To further investigate these relationships, the color correlation analysis was performed across all samples, where the size of the circles and the color indicate correlations red for negative and blue for positive correlation [41]. Correlation analysis of the strawberry pomace extract samples revealed strong positive relationships among TPC, TFC, and antioxidant assays (DPPH, ABTS, CUPRAC, FRAP), with all correlation coefficients exceeding 0.83 and significance at *p* < 0.001 (Figure 3).

The strongest correlations were observed between FRAP and CUPRAC (r = 0.9730) and DPPH and FRAP (r = 0.9572), indicating high consistency among different antioxidant capacity assays. Moderate positive correlations were found between TPC/TFC and MC ability as well as the PM assay, particularly with PM (r = 0.5517 for TPC; r = 0.3561 for TFC), suggesting that phenolics partially contribute to overall reducing power.

Weak or non-significant correlations were observed between antioxidant parameters and enzyme inhibition activities (AChE, BChE, tyrosinase, α-amylase, α-glucosidase). Notably, BChE showed a moderate negative correlation with α-glucosidase (r ≈ −0.4), which explains only a small portion of the variability (r^2^ ≈ 0.16). Therefore, this correlation should not be interpreted as evidence of a direct biochemical relationship, but rather as an observation reflecting differences in enzyme inhibition profiles across samples. These results demonstrate that antioxidant power is largely determined by the amount of phenolic and flavonoid compounds present, whereas these compounds do not appear to play a consistent or dominant role in enzyme inhibition. Based on the weak correlations observed, it is therefore likely that other phytochemical classes, including terpenoids, alkaloids, saponins, and minor secondary metabolites, contribute significantly to the inhibitory activities. Literature supports this interpretation; for example, alkaloid-rich fractions from *Aristotelia chilensis* leaf extracts have been shown to strongly inhibit both acetylcholinesterase (AChE) and butyrylcholinesterase (BChE) in vitro, highlighting the potential role of non-phenolic constituents in multi-enzyme inhibition [42]. While a detailed quantitative analysis of these individual compounds was not performed in this study, such compounds are known to interact with enzyme active or allosteric sites, thereby modulating enzymatic activity, and may account for the inhibitory effects observed in strawberry pomace extracts. Taken together, these findings highlight that the optimal extraction conditions must be considered in an activity-specific context, as a single set of parameters does not maximize all bioactivities simultaneously. This nuanced perspective reflects the complex phytochemical diversity of strawberry pomace and its implications for multifunctional bioactivity.

### 3.4. Principal Component Analysis (PCA)

**Figure 4:**
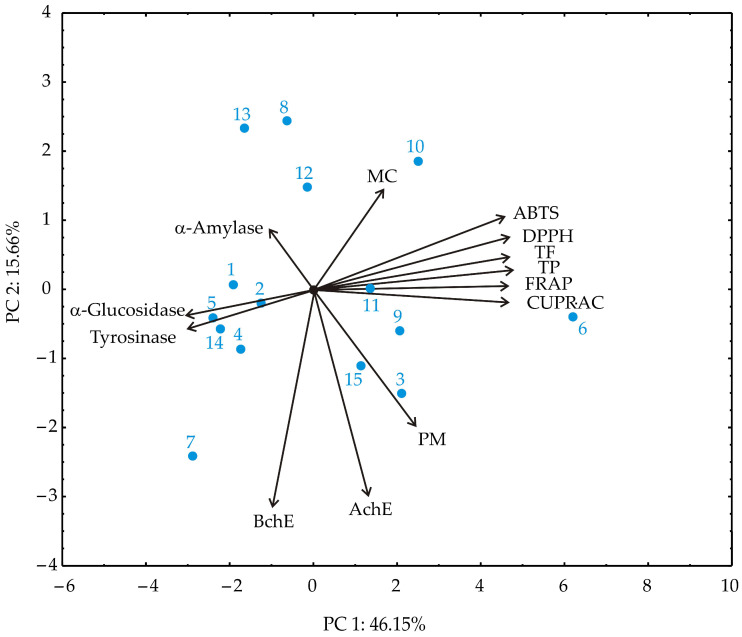
Principal component analysis (PCA) of strawberry pomace extracts samples based on observed responses.

Principal component analysis (PCA) of the obtained results revealed two principal components explaining 61.81% of the total variance (Figure 4). PC1 accounted for 46.15% of the variance and was primarily associated with antioxidant capacity and phenolic content. Variables including FRAP, CUPRAC, DPPH, TPC, TFC, and ABTS had the highest positive loadings on PC1, indicating strong collinearity and a dominant contribution to antioxidant potential. PC2 explained 15.66% of the variance and was characterized by high negative loadings from enzyme inhibitory activities, particularly BChE and AChE, as well as the PM assay. In contrast, MC and α-amylase contributed moderately and positively to PC2.

The PCA biplot clearly separated these two functional domains: antioxidant-related variables clustered together, pointing in the opposite direction from enzyme inhibition variables. This separation highlights the distinct biochemical profiles of the extracts and suggests that antioxidant and enzyme inhibitory effects are governed by different sets of phytochemicals.

In the PCA analysis, DPPH, ABTS, FRAP, CUPRAC, and TPC/TFC assays exhibited strong positive loadings on PC1, reflecting high collinearity among these radical scavenging and redox-based methods. In contrast, the phosphomolybdenum (PM) assay displayed a negative loading on PC2, co-occurring with high negative loadings for cholinesterase inhibition (AChE and BChE) and moderate positive contributions from metal chelation (MC) and α-amylase inhibition. This distribution indicates that PM captures aspects of antioxidant capacity complementary to, rather than redundant with, the radical scavenging or reducing power measured by the other assays. The divergence likely arises from the distinct redox mechanism and matrix-dependence of the PM assay, highlighting antioxidant features not fully represented by standard radical scavenging methods. These results underscore the importance of employing a diverse suite of assays to comprehensively characterize the bioactive potential of strawberry pomace extracts, encompassing multiple mechanisms of action and functional pathways.

### 3.5. Cluster Analysis

**Figure 5:**
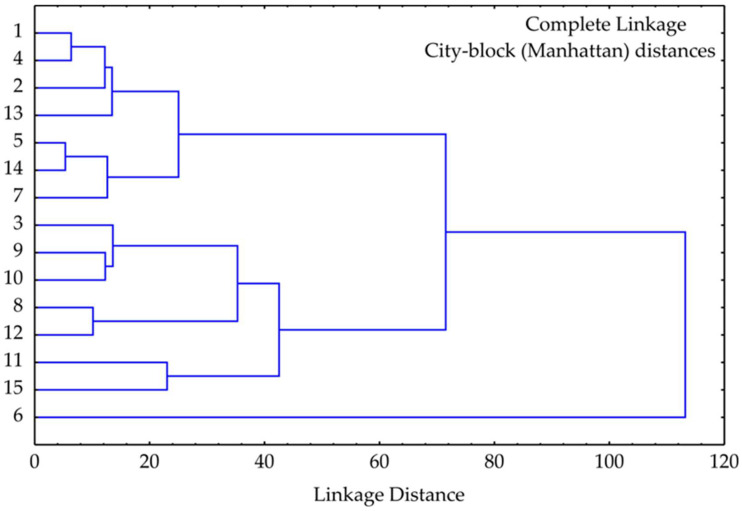
Cluster of strawberry pomace extract samples based on observed responses.

Hierarchical cluster analysis (HCA) of the strawberry extract samples, performed using complete linkage and City-block (Manhattan) distances, revealed three main clusters reflecting similarities in their bioactive profiles (Figure 5).

- Cluster I grouped samples 1, 2, 4, 5, 13, and 14, showing high similarity at low linkage distances. These samples likely share comparable antioxidant profiles and phenolic contents, consistent with PCA and correlation analysis. Notably, this cluster includes samples with the highest total phenolic content and antioxidant activity.
- Cluster II comprised samples 3, 7, 8, 9, 10, 11, 12, and 15, exhibiting moderate intra-cluster similarity. Sub-clusters such as (9, 10) and (8, 12) suggest close resemblance in bioactivity, reflecting intermediate levels of both antioxidant and enzyme inhibitory properties.
- Cluster III consisted solely of sample 6, which was separated from all others at a high linkage distance (>100), indicating a unique biochemical profile. This separation aligns with PCA results, where sample 6 exhibited extreme values along PC1, likely due to its exceptional antioxidant potency.

Generally, the HCA confirms the distinct biochemical and functional characteristics of sample 6 while highlighting similarities and variations among the remaining extracts.

### 3.6. Artificial Neural Networks

Artificial Neural Networks (ANNs) were developed to model the observed responses, including TPC, TFC, antioxidant assays (DPPH, ABTS, CUPRAC, FRAP, MC, and PM), and enzyme inhibition activities (AChE, BChE, Tyrosinase, α-amylase, α-glucosidase). Three separate ANN models were constructed (ANN1, ANN2, ANN3), with their architectures and parameters presented in Table 5, Table 6, Table 7, Table 8, Table 9, Table 10, Table 11, Table 12 and Table 13.

The optimal number of neurons in the hidden layers was determined to be 9, 10, and 10, corresponding to network structures MLP 3-9-2, MLP 3-10-6, and MLP 8-6-1/3-10-5 for ANN1, ANN2, and ANN3, respectively. Weight coefficients and biases for both input and output layers are listed in the corresponding tables.

The developed models demonstrated excellent predictive performance, with coefficients of determination (r^2^) reaching 0.999, 0.999, and 0.999 during the training phase, and 0.997, 0.996, and 0.999 during testing for ANN1, ANN2, and ANN3, respectively, confirming the validity and robustness of the models.

#### 3.6.1. ANN1

**Figure 6:**
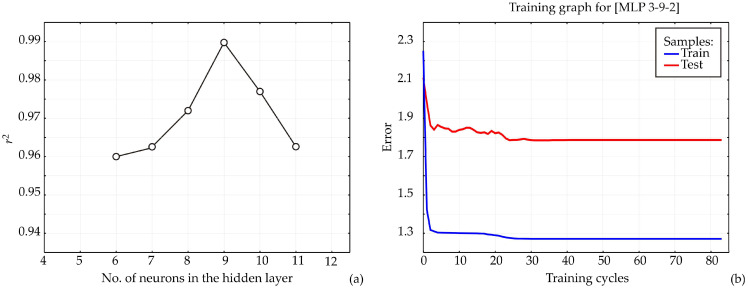
ANN1 calculation: (**a**) The dependence of the r^2^ value of the number of neurons in the hidden layer in the ANN1 model, (**b**) training results per epoch.

The ANN architecture, including bias terms and weight coefficients, is highly dependent on the initial assumptions of the matrix parameters, which are crucial for network construction and accurate fitting to the experimental data [43]. Additionally, variations in the number of neurons within the hidden layer can influence the model performance [44]. Therefor in this research, each network topology was executed 100,000 times to minimize the impact of random correlations resulting from initial parameter assumptions and weight initialization. In this way, the highest r^2^ value during the training cycle was achieved with nine neurons in the hidden layer (Figure 6a).

The model was trained over 100 epochs, and the training outcomes, namely, training accuracy and loss, are shown in Figure 6b. Training accuracy improved steadily as the number of epochs increased, reaching an approximately stable value around the 70th to 80th epoch. Maximum training accuracy and minimum loss were observed within this range. Beyond the 80th epoch, minor fluctuations in accuracy and loss suggested the onset of overfitting. Therefore, training for 70 epochs is sufficient to attain high model accuracy while avoiding overfitting, whereas extending training beyond 80 epochs may compromise model generalization. The training accuracy plateaued between the 70th and 80th epoch for all models. Beyond this point, accuracy increased only marginally while loss continued to decrease, a characteristic sign of overfitting. To prevent this, the model was restricted to 70 training epochs, a stage at which the curves still indicate stable learning without divergence. Thus, the final model was explicitly selected before significant overfitting occurred.

The weight coefficients and biases *W*_1_ and *B*_1_ used in the ANN1 modelling for input parameters (time, temperature and plant to solvent ratio) and *W*_2_ and *B*_2_ used for calculation within the ANN1 model for output parameters TPC and TFC throughout training, testing and validation steps are given in Table 6 and Table 7.

#### 3.6.2. ANN2

The weight coefficients and biases W_1_ and B_1_ used in the ANN2 modelling for input parameters (time, temperature and plant to solvent ratio) and W_2_ and B_2_ used for calculation within the ANN2 model for output parameters DPPH, ABTS, CUPRAC, FRAP, MC, PM throughout training, testing and validation steps are given in Table 9 and Table 10.

#### 3.6.3. ANN_3_

The weight coefficients and biases W_1_ and B_1_ used in the ANN_3_ modelling for input parameters (time, temperature and plant to solvent ratio) and W_2_ and B_2_ used for calculation within the ANN_3_ model for output parameters AChE, BChE, Tyrosinase, α-amylase and α-glucosidase throughout training, testing and validation steps are given in Table 12 and Table 13.

### 3.7. Model Validation

To assess the accuracy of the developed ANN models, several performance metrics were evaluated, including reduced chi-square (χ^2^), root mean square error (RMSE), mean bias error (MBE), mean percentage error (MPE), total squared error (SSE), average absolute relative deviation (AARD), and coefficient of determination (r^2^) (Table 14). Evaluation of these metrics indicated only minor lack-of-fit errors, suggesting that the ANN models satisfactorily predicted the values of TPC, TFC, antioxidant assays (DPPH, ABTS, CUPRAC, FRAP, MC and PH), and enzyme inhibition activities (AChE, BChE, Tyrosinase, α-amylase, and α-glucosidase).

### 3.8. ANN Optimization and Standard Score Analysis

The optimization of the ANN outputs was performed using the results presented in Table 6, Table 7, Table 8, Table 9, Table 10, Table 11, Table 12 and Table 13, according to Equation (1). The aim was to optimize TPC, TFC, antioxidant activities (DPPH, ABTS, CUPRAC, FRAP, MC, and PM), and enzyme inhibition activities (AChE, BChE, Tyrosinase, α-amylase, α-glucosidase) of strawberry pomace extracts. The input variables considered in the ANN models were extraction time, temperature, and plant material type.

Optimization was carried out using ANN1, ANN2, and ANN3, with the number of generations reaching 88, 69, and 84, respectively. The population size was set to 100 for each input variable, and 50 points were selected on the Pareto front. The ANN models consistently identified sample 6 as the optimal extract.

**Figure 7:**
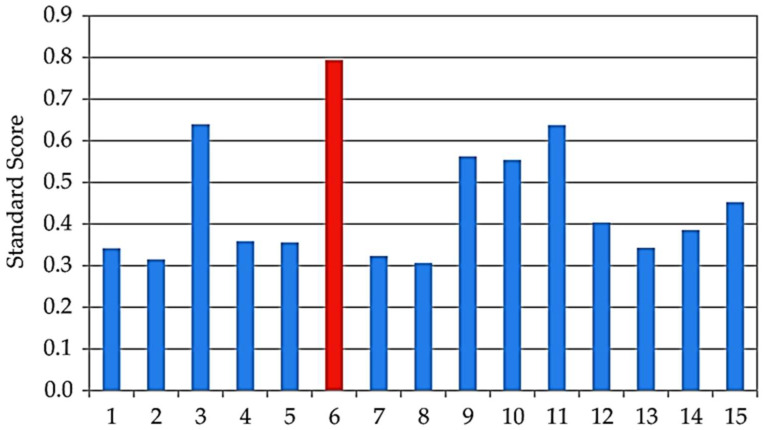
Z-score analysis of the observed samples 1–15.

Validation of the ANN optimization was performed using Standard Score analysis, where the mean Z-score across all measured responses was calculated as described by Malešević et al. [45]. Sample 6 achieved the highest combined Z-score of 0.794, confirming it as the optimal condition (Figure 7). This extract represents the best compromise between antioxidant and enzyme inhibition activities, supporting the effectiveness of ANN-based optimization in guiding process parameters.

The ANN model optimization and Standard Score analysis identified sample 6 as the optimal extract. This sample was obtained using 20 min UAE at 50 °C, with a sample-to-solvent ratio of 1:20 g/mL. The measured values for this optimal strawberry pomace extract were as follows:

- Total phenolics (TP): 16.494 ± 0.149 mg GAE/g
- Total flavonoids (TF): 2.103 ± 0.006 mg RE/g
- Antioxidant assays:
  DPPH 32.695 ± 0.568 mg TE/g,
  ABTS 46.764 ± 0.315 mg TE/g,
  CUPRAC 57.285 ± 1.619 mg TE/g,
  FRAP 38.900 ± 1.298 mg TE/g,
  MC 19.140 ± 0.148 mg EDTAE/g,
  PM 0.542 ± 0.032 mmol TE/g
- Enzyme inhibition assays:
  AChE 2.320 ± 0.019 mg GALAE/g,
  BChE 2.058 ± 0.016 mg GALAE/g,
  Tyrosinase 55.453 ± 0.201 mg KAE/g,
  α-amylase 0.738 ± 0.019 mmol ACAE/g,
  α-glucosidase 0.965 ± 0.085 mmol ACAE/g.

## 4. Conclusions

Ultrasound-assisted extraction (UAE) proved to be an efficient and sustainable approach for the valorization of strawberry pomace, yielding extracts enriched with bioactive compounds. While a strong positive correlation was observed between total phenolic content and antioxidant potential, enzyme inhibitory activities exhibited a more complex and less direct relationship with total phenols. Instead, these activities were influenced by distinct phytochemical fractions, likely including terpenoids, alkaloids, saponins, and other minor secondary metabolites. This divergence highlights the complexity of the extract composition and clearly demonstrates that total phenolics are insufficient to predict multifunctional enzyme inhibitory effects. Multivariate statistical analyses and artificial neural network (ANN) modeling further confirmed the effectiveness of integrating advanced predictive tools with UAE, identifying optimal extraction conditions (20 min at 50 °C and a 1:20 g/mL plant-to-solvent ratio) that balance antioxidant and enzyme inhibitory activities. These findings not only reinforce the value of the UAE for obtaining phenolic-rich antioxidant fractions but also reveal the significant and previously underexplored contribution of non-phenolic compounds to enzyme inhibition, representing a novel insight into the functional valorization of agri-food by-products.

Future studies will focus on detailed chromatographic characterization of the extracts using advanced chromatographic techniques to enable the identification and quantitative determination of individual bioactive compounds responsible for enzyme inhibition. Such analyses will allow validation of the optimization model by directly linking specific molecular features and chromatographic peaks to distinct functional domains (antioxidant activity versus enzyme inhibition). Moreover, compound-level characterization will significantly enhance the reproducibility, application value, and scalability of the UAE process, thereby providing a robust foundation for bioavailability assessment and the development of food, nutraceutical, and pharmaceutical formulations.

## Figures and Tables

**Table 1:** Experimental design for ultrasound-assisted extraction of strawberry pomace.

Sample	Time [min]	Temperature [°C]	Sample to Solvent Ratio [g/mL]
1	10	25	10
2	10	50	15
3	10	75	20
4	20	25	15
5	20	50	10
6	20	50	20
7	20	75	15
8	30	25	20
9	30	50	10
10	30	50	15
11	30	75	10
12	10	25	20
13	20	25	10
14	10	75	15
15	30	75	20

**Table 2:** Total phenolic and total flavonoid content ^1^ of strawberry pomace extracts under different experimental conditions.

Samples	TPC (mg GAE/g) ^2^	TFC (mg RE/g) ^3^
1	11.184 ± 0.103 ^de^	0.804 ± 0.034 ^a^
2	10.793 ± 0.159 ^de^	1.001 ± 0.025 ^cd^
3	13.301 ± 0.473 ^g^	1.648 ± 0.063 ^hj^
4	10.589 ± 0.070 ^cd^	0.858 ± 0.048 ^ab^
5	9.372 ± 0.358 ^ab^	1.023 ± 0.046 ^de^
6	16.494 ± 0.149 ^i^	2.103 ± 0.006 ^i^
7	8.692 ± 0.507 ^a^	0.880 ± 0.049 ^abc^
8	10.970 ± 0.191 ^de^	1.139 ± 0.020 ^ef^
9	14.955 ± 0.328 ^hj^	1.563 ± 0.042 ^h^
10	15.213 ± 0.316 ^j^	1.617 ± 0.026 ^h^
11	12.236 ± 0.224 ^f^	1.765 ± 0.043 ^j^
12	11.475 ± 0.026 ^ef^	1.263 ± 0.075 ^fg^
13	9.982 ± 0.425 ^bc^	1.180 ± 0.037 ^fg^
14	9.174 ± 0.071 ^a^	0.952 ± 0.050 ^bcd^
15	14.176 ± 0.286 ^h^	1.295 ± 0.026 ^g^

^1^ values are means ± SD of three measurements; mean values within each column with different letters (a–j) differ significantly (*p* ≤ 0.05). ^2^ mg gallic acid equivalent/g. ^3^ mg rutin equivalent/g.

**Table 3:** Antioxidant activity ^1^ of strawberry pomace extracts under different extraction conditions.

Samples	DPPH (mg TE/g) ^2^	ABTS (mg TE/g) ^2^	CUPRAC (mg TE/g) ^2^	FRAP (mg TE/g) ^2^	MC (mg EDTAE/g) ^3^	PM (mmol TE/g) ^4^
1	16.634 ± 0.521 ^ab^	21.335 ± 0.432 ^b^	31.314 ± 0.276 ^abc^	17.485 ± 0.044 ^a^	16.389 ± 0.610 ^cd^	0.532 ± 0.028 ^cde^
2	17.709 ± 0.509 ^bc^	22.707 ± 0.071 ^bc^	33.100 ± 0.110 ^c^	21.161 ± 0.217 ^d^	17.456 ± 0.502 ^cdef^	0.441 ± 0.012 ^abc^
3	27.054 ± 0.144 ^h^	34.898 ± 0.937 ^g^	43.283 ± 0.530 ^ef^	28.328 ± 0.131 ^h^	14.004 ± 0.586 ^ab^	0.591 ± 0.014 ^e^
4	17.635 ± 0.593 ^bc^	22.765 ± 0.497 ^bc^	31.747 ± 0.894 ^bc^	17.776 ± 0.045 ^ab^	15.740 ± 0.253 ^bc^	0.522 ± 0.020 ^cde^
5	15.530 ± 0.576 ^a^	15.503 ± 0.280 ^a^	29.701 ± 0.261 ^a^	19.310 ± 0.120 ^c^	18.501 ± 1.477 ^efg^	0.440 ± 0.050 ^abc^
6	32.695 ± 0.568 ^i^	46.764 ± 0.315 ^i^	57.285 ± 1.619 ^g^	38.900 ± 1.298 ^i^	19.140 ± 0.148 ^fg^	0.542 ± 0.032 ^de^
7	16.261 ± 0.428 ^a^	15.380 ± 0.649 ^a^	30.010 ± 0.354 ^ab^	17.596 ± 0.432 ^ab^	12.929 ± 0.577 ^a^	0.536 ± 0.022 ^de^
8	22.457 ± 0.368 ^f^	32.279 ± 0.265 ^f^	32.648 ± 0.859 ^c^	22.445 ± 0.473 ^de^	17.188 ± 0.293 ^cde^	0.394 ± 0.021 ^a^
9	23.886 ± 0.708 ^g^	32.856 ± 0.768 ^f^	43.669 ± 0.903 ^f^	28.556 ± 0.313 ^h^	17.971 ± 0.005 ^def^	0.594 ± 0.005 ^e^
10	26.829 ± 0.206 ^h^	38.387 ± 0.758 ^h^	42.129 ± 0.608 ^ef^	28.546 ± 0.434 ^h^	18.001 ± 0.452 ^def^	0.517 ± 0.001 ^bcde^
11	22.619 ± 0.579 ^fg^	26.854 ± 0.155 ^d^	42.861 ± 0.406 ^ef^	26.130 ± 0.410 ^g^	20.374 ± 0.283 ^g^	0.580 ± 0.019 ^e^
12	21.935 ± 0.581 ^ef^	29.346 ± 1.007 ^e^	35.250 ± 0.255 ^d^	22.747 ± 0.262 ^e^	17.474 ± 0.827 ^cdef^	0.482 ± 0.034 ^abcd^
13	19.428 ± 0.589 ^d^	23.197 ± 0.167 ^c^	29.870 ± 0.108 ^ab^	19.527 ± 0.600 ^c^	18.489 ± 0.900 ^ef^	0.424 ± 0.060 ^ab^
14	17.980 ± 0.471 ^c^	16.036 ± 0.397 ^a^	30.639 ± 0.252 ^ab^	19.027 ± 0.488 ^bc^	18.109 ± 0.440 ^def^	0.461 ± 0.049 ^abcd^
15	21.100 ± 0.818 ^e^	22.209 ± 0.203 ^bc^	41.530 ± 0.451 ^e^	24.499 ± 0.212 ^f^	8.611 ± 0.503 ^efg^	0.74 ± 0.035 ^f^

^1^ values are means ± SD of three measurements; mean values (a–i) differ significantly (*p* ≤ 0.05). ^2^ mg Trolox equivalent/g. ^3^ mg EDTA equivalent/g. ^4^ mmol Trolox equivalent/g /g.

**Table 4:** Enzyme inhibitor activity ^1^ of strawberry pomace extracts under different extraction conditions.

Samples	AChE (mg GALAE/g) ^2^	BChE (mg GALAE/g) ^2^	Tyrosinase (mg KAE/g) ^3^	α-Amylase (mmol ACAE/g) ^4^	α-Glucosidase (mmol ACAE/g) ^4^
1	1.973 ± 0.077 ^de^	1.755 ± 0.201 ^bcde^	52.258 ± 1.345 ^bcd^	0.833 ± 0.010 ^bcdef^	1.070 ± 0.066 ^cd^
2	1.926 ± 0.046 ^de^	2.094 ± 0.152 ^fgh^	54.097 ± 0.383 ^def^	0.739 ± 0.026 ^a^	0.826 ± 0.019 ^abc^
3	2.540 ± 0.020 ^h^	1.789 ± 0.089 ^cdef^	54.720 ± 0.516 ^ef^	0.863 ± 0.008 ^def^	1.114 ± 0.100 ^d^
4	1.948 ± 0.058 ^de^	2.141 ± 0.045 ^gh^	53.661 ± 0.405 ^cdef^	0.785 ± 0.022 ^abc^	1.090 ± 0.042 ^d^
5	2.213 ± 0.060 ^fg^	2.078 ± 0.103 ^efgh^	52.503 ± 0.733 ^bcde^	0.862 ± 0.021 ^def^	0.924 ± 0.015 ^abcd^
6	2.320 ± 0.019 ^fg^	2.058 ± 0.016 ^efgh^	55.453 ± 0.201 ^f^	0.738 ± 0.019 ^a^	0.965 ± 0.085 ^abcd^
7	2.242 ± 0.028 ^fg^	2.360 ± 0.044 ^h^	54.470 ± 0.892 ^def^	0.852 ± 0.012 ^cdef^	0.864 ± 0.119 ^abcd^
8	1.579 ± 0.085 ^a^	1.545 ± 0.056 ^abcd^	52.718 ± 0.571 ^bcde^	0.827 ± 0.060 ^bcde^	0.742 ± 0.086 ^ab^
9	1.996 ± 0.048 ^e^	2.160 ± 0.134 ^gh^	54.086 ± 0.420 ^def^	0.816 ± 0.033 ^bcd^	0.706 ± 0.086 ^a^
10	1.817 ± 0.030 ^bd^	1.388 ± 0.014 ^a^	53.475 ± 0.772 ^cdef^	0.827 ± 0.008 ^bcde^	0.988 ± 0.125 ^bcd^
11	2.194 ± 0.025 ^f^	1.904 ± 0.097 ^efg^	52.305 ± 0.475 ^bcd^	0.902 ± 0.019 ^f^	1.123 ± 0.018 ^d^
12	1.881 ± 0.032 ^bde^	1.498 ± 0.139 ^abc^	50.553 ± 0.615 ^b^	0.844 ± 0.008 ^cdef^	1.016 ± 0.082 ^cd^
13	1.740 ± 0.087 ^b^	1.451 ± 0.080 ^ab^	51.374 ± 0.663 ^bc^	0.895 ± 0.009 ^ef^	1.019 ± 0.153 ^cd^
14	2.357 ± 0.070 ^g^	1.994 ± 0.075 ^efg^	52.666 ± 0.559 ^bcde^	0.889 ± 0.012 ^ef^	0.929 ± 0.082 ^abcd^
15	2.170 ± 0.020 ^f^	1.847 ± 0.138 ^defg^	43.139 ± 1.983 ^a^	0.766 ± 0.021 ^ab^	0.902 ± 0.063 ^abcd^

^1^ values are means ± SD of three measurements; mean values (a–h) differ significantly (*p* ≤ 0.05). ^2^ mg galantamine equivalent/g. ^3^ mg kojic acid equivalent/g. ^4^ mmol acarbose equivalent/g.

**Table 5:** Artificial Neural Network model summary (performance and errors), for training, and testing cycles for TPC and TFC.

Net. Name	Train Perf.	Test Perf.	Valid Perf.	Train Error	Test Error	Valid Error	Training Algorithm	Error Function	Hidden Activation	Output Activation
MLP 3-9-2	0.999	0.997	0.995	1.276	1.778	1.969	BFGS 122	SOS	Tanh	Identity

**Table 6:** The weight coefficients and biases W_1_ and B_1_ for ANN_1_.

	1	2	3	4	5	6	7	8	9
Time	1.829	0.046	2.049	−0.616	2.110	−2.953	−2.765	3.182	−1.494
Temperature	0.727	−4.094	−1.845	−0.996	−2.036	−0.604	−0.210	−0.819	0.609
Plant to solvent ratio	1.245	1.314	−2.820	−0.821	−2.044	−0.676	0.034	−2.235	−1.004
Bias	−2.096	−1.565	1.896	0.695	1.592	1.396	0.093	1.054	0.359

Time

1.829

0.046

2.049

−0.616

2.110

−2.953

−2.765

3.182

−1.494

Temperature

0.727

−4.094

−1.845

−0.996

−2.036

−0.604

−0.210

−0.819

0.609

Plant to solvent ratio

1.245

1.314

−2.820

−0.821

−2.044

−0.676

0.034

−2.235

−1.004

Bias

−2.096

−1.565

1.896

0.695

1.592

1.396

0.093

1.054

0.359

**Table 7:** The weight coefficients and biases W_2_ and B_2_ for ANN1.

	1	2	3	4	5	6	7	8	9	Bias
TPC	0.182	−1.142	−0.886	0.898	1.159	−1.269	1.300	−0.560	−0.347	0.433
TFC	1.034	−0.573	−1.155	0.790	1.744	−0.006	−0.670	−1.067	0.513	−0.045

**Table 8:** Artificial Neural Network model summary (performance and errors), for training, and testing cycles for DPPH, ABTS, CUPRAC, FRAP, MC, and PM.

Net. Name	Train Perf.	Test Perf.	Valid Perf.	Train Error	Test Error	Valid Error	Training Algorithm	Error Function	Hidden Activation	Output Activation
MLP 3-10-6	0.999	0.996	0.993	1.233	1.555	2.999	BFGS 10000	SOS	Tanh	Tanh

**Table 9:** The weight coefficients and biases W_1_ and B_1_ for ANN_2_.

	1	2	3	4	5	6	7	8	9	10
Time	−14.977	3.657	2.872	0.461	6.655	−18.666	−0.520	−10.250	−7.010	−0.021
Temperature	3.018	0.938	1.333	−4.795	3.146	11.988	−1.591	−2.983	−8.548	−3.227
Plant to solvent ratio	10.497	0.918	3.496	5.038	0.083	2.971	−0.142	−3.607	6.351	3.393
Bias	0.137	−1.069	−3.615	3.482	−9.731	−1.995	2.863	6.613	1.041	−0.487

**Table 10:** The weight coefficients and biases W_2_ and B_2_ for ANN_2_.

	1	2	3	4	5	6	7	8	9	10	Bias
DPPH	4.276	−3.903	−2.660	−1.743	−1.045	−3.002	0.357	−3.551	−5.818	5.456	1.742
ABTS	4.110	−4.917	−2.961	−1.942	−1.443	−2.877	−0.072	−4.084	−6.452	6.106	2.459
CUPRAC	4.456	−3.557	−2.846	−1.574	−0.472	−3.038	0.827	−3.632	−5.526	5.181	1.431
FRAP	3.428	−1.782	−1.663	−0.924	0.568	−2.270	3.905	−2.277	−3.581	3.165	−1.614
MC	−0.251	−2.107	−0.451	−4.768	0.937	0.167	2.405	−0.776	−1.791	1.581	4.779
PM	1.252	4.388	0.960	4.212	7.144	−0.845	3.328	1.751	2.877	−2.812	−1.782

**Table 11:** Artificial Neural Network model summary (performance and errors), for training, and testing cycles for AChE, BChE, Tyrosinase, α-amylase, and α-glucosidase.

Net. Name	Train Perf.	Test Perf.	Valid Perf.	Train Error	Test Error	Valid Error	Training Algorithm	Error Function	Hidden Activation	Output Activation
MLP 3-10-5	0.999	0.999	0.995	1.688	1.998	2.999	BFGS 299	SOS	Log	Iden

**Table 12:** The weight coefficients and biases W_1_ and B_1_ for ANN_3_.

	1	2	3	4	5	6	7	8	9	10
Time	−3.656	−3.576	−3.421	−8.473	−2.593	−2.336	−4.079	−1.876	−2.826	−6.256
Temperature	3.569	3.666	3.869	7.991	5.218	6.572	5.171	9.862	5.198	13.951
Plant to solvent ratio	−8.416	0.785	−4.395	0.029	0.998	−6.147	−0.617	−2.914	−5.130	−4.205
Bias	1.173	2.266	0.951	−1.431	0.198	−1.555	−1.709	−3.460	−1.273	0.145

**Table 13:** The weight coefficients and biases W2 and B2 for ANN3.

	1	2	3	4	5	6	7	8	9	10	Bias
AChE	−0.027	1.432	−0.714	−2.462	−0.700	2.516	4.286	−1.351	−2.748	0.838	−0.399
BChE	−2.386	3.737	−1.091	−0.667	−3.849	2.436	0.886	−2.347	0.905	3.006	−0.461
Tyrosinase	−2.412	−0.178	2.002	−2.384	0.145	1.909	2.855	−2.332	−0.977	0.718	0.810
α-amylase	2.507	−2.035	0.593	−0.135	2.128	1.838	1.083	1.898	−4.851	−2.718	0.894
α-glucosidase	−2.729	2.291	3.296	−3.347	−0.453	−2.418	2.054	0.033	2.884	−0.795	−0.695

**Table 14:** The “goodness of fit” for the observed ANN models.

	χ^2^	RMSE	MBE	MPE	SSE	AARD	r^2^
TPC	0.007	0.078	0.004	0.557	0.091	0.975	0.999
TFC	0.000	0.009	0.000	0.666	0.001	0.160	0.999
DPPH	0.019	0.129	0.014	0.545	0.248	2.507	0.999
ABTS	0.097	0.291	0.114	1.064	1.072	3.342	0.999
CUPRAC	0.061	0.230	0.026	0.492	0.783	2.841	0.999
FRAP	0.027	0.154	0.019	0.572	0.349	2.356	0.999
MC	0.003	0.050	0.008	0.244	0.037	1.069	0.999
PM	0.000	0.003	−0.001	0.537	0.000	0.066	0.999
AChE	0.000	0.008	0.002	0.353	0.001	0.137	0.999
BChE	0.000	0.010	0.001	0.485	0.001	0.135	0.999
Tyrosinase	0.005	0.068	−0.016	0.112	0.065	0.994	0.999
α-amylase	0.000	0.001	0.000	0.142	0.000	0.018	0.999
α-glucosidase	0.000	0.005	0.000	0.402	0.000	0.054	0.999

## Data Availability

The original contributions presented in this study are included in the article/Supplementary Material. Further inquiries can be directed to the corresponding author.

## Supplementary Materials

The following supporting information can be downloaded at: https://www.mdpi.com/article/10.3390/antiox15010050/s1, Table S1: AGREEprep Analytical Greeness Metric for Sample Preparation (lyophilization and extraction of strawberry pomace).

## Abbreviations

The following abbreviations are used in this manuscript:

AARD	Absolute average relative deviation
ABTS	2.2′-azino-bis (3-ethylbenzothiazoline-6-sulphonic acid)
ACAE	Acarbose equivalent
AChE	Acetylcholinesterase
ANN	Artificial neural network
BChE	Butyrylcholinesterase
CAGR	Compound Annual Growth Rate
CE	Catechin equivalents
CUPRAC	Cupric reducing antioxidant capacity
DPPH	2.2-diphenyl-1-picryl-hydrazyl-hydrate
EDTA	Ethylenediaminetetraacetic acid equivalents
FRAP	Ferric reducing antioxidant power
GAE	Gallic acid equivalents
GALAE	Galantamine equivalents
HCA	Hierarchical cluster analysis
KAE	Kojic acid equivalents
MBE	Mean bias error
MC	Metal chelating assay
MLP	Multi-layer perceptron
MPE	Mean percentage error
PCA	Principal Component Analysis
PM	Phosphomolybdenum assay
RMSE	Root mean square error
RE	Rutin equivalents
SSE	Sum of square error
TE	Trolox equivalents
TFC	Total flavonoids content
TPC	Total phenolics content
UAE	Ultrasound assisted extraction
UVA	Ultraviolet A
